# MWAHCA: A Multimedia Wireless Ad Hoc Cluster Architecture

**DOI:** 10.1155/2014/913046

**Published:** 2014-03-11

**Authors:** Juan R. Diaz, Jaime Lloret, Jose M. Jimenez, Sandra Sendra

**Affiliations:** Universidad Politécnica de Valencia, Camino Vera, s/n 46022 Valencia, Spain

## Abstract

Wireless Ad hoc networks provide a flexible and adaptable infrastructure to transport data over a great variety of environments. Recently, real-time audio and video data transmission has been increased due to the appearance of many multimedia applications. One of the major challenges is to ensure the quality of multimedia streams when they have passed through a wireless ad hoc network. It requires adapting the network architecture to the multimedia QoS requirements. In this paper we propose a new architecture to organize and manage cluster-based ad hoc networks in order to provide multimedia streams. Proposed architecture adapts the network wireless topology in order to improve the quality of audio and video transmissions. In order to achieve this goal, the architecture uses some information such as each node's capacity and the QoS parameters (bandwidth, delay, jitter, and packet loss). The architecture splits the network into clusters which are specialized in specific multimedia traffic. The real system performance study provided at the end of the paper will demonstrate the feasibility of the proposal.

## 1. Introduction

Wireless ad hoc networks are formed by a set of distributed nodes inside a limited geographic area. Generally, the physical topology is given by the density, the placement of the nodes, and their mobility along the time in the area. Wireless nodes are able to communicate without a wired infrastructure by using an ad hoc communication. The way they are organized in the physical topology and the way they communicate with other nodes determine the logical network topology and the routes followed by the data in the ad hoc network [1]. Once the logical topology is built, end nodes have the needed transport infrastructure for the information exchange. This infrastructure allows monitoring, remote management, data gathering, and so forth. There could be a huge number of logical topologies depending on the criteria and algorithms followed by the nodes when the network is built [2]. The architecture should take into account the network traffic features with the objective of optimizing the performance and efficiency of the transmissions.

Cluster-based architectures are very common topologies in ad hoc networks. These architectures organize the nodes in small groups of nodes that work independently and autonomously [3]. Nodes in each cluster can communicate with other nodes establishing neighborhoods or through the neighborhoods of their neighbors. At the same time, a cluster can communicate with other clusters or with external networks through a higher hierarchical level, which is shared by all clusters [4]. We can find in the related literature many wireless ad hoc clustering algorithms and schemes. The network topology will depend on the neighbor selection criteria when building the cluster. Moreover, the motion of the nodes change the topology constantly, which increases the number of management messages [5]. Neighbor node selection has been widely researched in many network structures [6]. Depending on the purpose nodes can be organized taking into account the geographic distance [4], decrease the energy consumption [7], decrease convergence time, maximize the whole available bandwidth for data transmission, fault tolerance, or load distribution purposes, and so forth.

Due to the fast growth and development of wireless technologies, wireless ad hoc networks are becoming more and more common between people. There are many emergent applications and new environments to be used. New tendencies are user-oriented and service-oriented wireless ad hoc networks [8], where one of the main ones is the real-time audio and video streaming (sometimes their use in other types of networks is very complex or too expensive).

In order to deliver real time data traffic inside a wireless ad hoc network, it is essential to quantify and measure the network quality of service (QoS) parameters: delay, jitter, lost packets, and guaranteed bandwidth [9]. QoS should be analyzed from two complementary points of view. On one hand, it should be taken into account that for each multimedia stream, QoS parameters should be kept inside a range along the time. On the other hand, different multimedia streams (depending on whether audio or video, or depending on the used codec) will have different optimum QoS values and ranges.

Although there is some interest to manage the traffic flow in ad hoc networks [10], many researchers take into account QoS parameters in their proposals [11]. But existing cluster systems do not use QoS parameters as criteria to build the logical topology. So, the obtained QoS values could be outside of the required range. Therefore, the quality of the multimedia communications will not be guaranteed and the quality of the experience (QoE) of the end users will be affected. Moreover, QoS parameters should be monitored continuously because their values may vary considerably due to network changes (node joining and leavings, new concurrent audio, video, and data streams).

In this paper we propose a new architecture to build wireless ad hoc clusters based on QoS parameters criteria. The architecture allows structuring the network taking into account the features of the multimedia streaming, the number of streams delivered through the network, and the capacity of the nodes belonging to the network. The objective is to offer a guaranteed and differentiated service for each multimedia stream in order to optimize the communication between nodes and taking full advantage of the available bandwidth, but guaranteeing the required delay, jitter, and packet loss levels.

The remainder of the paper is structured as follows.  Section 2 reviews the papers we have found related to the multimedia streaming in ad hoc networks. In Section 3, we detail the proposed architecture, introduce the multimedia init profile (MIP) concept, and describe the most important processes of the architecture. The state machine of the architecture is described in Section 4.  Section 5 shows the performance study of the architecture in order to validate our proposal. Finally, Section 6 draws the conclusions and future work.

## 2. Related Works

In the recent years, the research on multimedia distribution over ad hoc networks has been increased. It has happened because of the improvement of the hardware capabilities and the appearance of new multimedia services. In order to support real-time multimedia applications, we should mainly take into account QoS constraints.

In [12], Zhang et al. propose several improvements over MAC protocols to solve some of the main problems as the stringent quality of service (QoS) requirements of video traffic, the limited wireless channel bandwidth, and the broadcast nature of wireless medium in ad hoc networks. Authors proposed two conflict avoidance strategies for reservation and contention interleaved wireless systems. With a dual buffer, the video packets being transmitted using contention or reservation-based channel access are separated and stored in two buffers. Authors also developed analytical models considering the interactions of reservation and contention periods. The performance tests were focused on the contention-based access. Simulation results showed that the backoff strategy can achieve higher throughput when the number of reserved periods in each superframe is large. Authors also checked the results of their proposal when transferring MPEG-4 video streams. The proposed hybrid approach with the two buffering architectures provided a considerably better performance, due to the higher reservation utilization and lower contention level.

Mehta and Narmawala used a video traffic model to generate video traffic frames in [13]. They observed that network coding performs well in lossy wireless ad hoc networks in both multicast and broadcast scenarios. Even in wireless ad hoc networks with low density of nodes network coding performs well using their multicopy packet transmission scheme. In their work, each sender node encodes the packet using a variant of network coding, which is random linear network coding (RLNC) with multigeneration mixing (MGM), with the aim to provide more protection to I (intraframe of MPEG 4 video traffic) frames in order to minimize the multiplicative loss by incurring slight delay in transmission. Mixing different types of packets increases the packet delivery fraction and reduces packet drop rate and block delay of multimedia transmission over wireless ad hoc networks.

Since the main weight of maintaining a fast multimedia delivery and an optimum path for the streams in an ad hoc network is carried out by the routing protocols [14], most authors have studied the routing protocols in ad hoc networks in order to know their features and which ones are the most appropriate to provide QoS [15]. Moreover, some authors have developed QoS-aware routing protocols for ad hoc networks such as the following ones.

Al Turki and Mehmood in [16] studied video streaming applications over ad hoc networks and analyzed the results obtained through simulations using the OPNET software. They also surveyed the main challenges in ad hoc network research and reviewed the QoS literature for ad hoc networks. They evaluated performance of video streaming applications over ad hoc networks by simulating few scenarios with 5 different routing protocols. The results show that it is possible to support multimedia applications over medium sized networks.

Jamali et al. demonstrated in [17] that ad hoc networks can support video streaming. In order to do it, they analyzed some routing protocols through simulations in OPNET environment in terms of multimedia and real-time application and QoS. In their study, they analyze AODV, DSR, OLSR, TORA, and GRP for multimedia streaming. Their results demonstrate that ad hoc networks can have good video streaming quality. They conclude the paper stating that designing a multimedia ad hoc network is difficult because of the higher QoS requirement and the kind of network topology.

Abdrabou and Zhuang presented in [18] a model-based quality-of-service (QoS) routing scheme for IEEE 802.11 ad hoc networks. This proposal is based on a cross-layer design approach. The scheme proposed selects the routes based on a geographical on-demand ad hoc routing protocol and checks the availability of the network resources by using traffic source and link-layer channel modeling. The system also considers the IEEE 802.11 features and the node interactions. The protocol checks if the selected route is able to admit traffic flow without affecting other flows already in service. The simulation results show that the proposal is efficient in resource utilization while satisfying the delay bound probabilistically with a low overhead.

Kandris et al. present in [19] a dual scheme based on the combined use of an energy aware hierarchical routing protocol with an intelligent video packet scheduling algorithm for efficient video communication, which aims at both energy saving and high QoS attainment. PEMuR adopts a routing protocol which is able to select the most energy efficient routing paths while it manages the network load according to the energy residues of the nodes and prevents useless data transmissions through the proposed use of an energy threshold. In addition, this protocol is able to reduce the video transmission rate with the minimum possible increase of distortion. The simulations performed by authors showed that this proposal prolongs the node lifetime. It also enhances the metric of network performance in the case of nodes with nonuniform energy distribution while maintaining high levels of the perceived video quality (PSNR).

Taing et al. [20] propose a routing scheme for multimedia services, which selects the shortest path by using power level. This proposed scheme selects the shortest path for multimedia traffic by applying larger power level because the delay is sensitive to such kind of traffic. Moreover, for nonreal time traffic, this algorithm uses smaller power level longer path for non-real time. They conclude that their proposal provides the lower mean number of hops for multimedia traffic than the mean number of hops for non-real-time traffic. As a result, the transmission delay of multimedia traffic can be decreased. They also show that its proposal scheme can provide higher throughput for multimedia traffic.

In [21] we presented multimedia-oriented architecture and protocol for wireless ad hoc networks. This proposal takes into account the multimedia services offered by the nodes in the wireless ad hoc network in order to select the best multimedia service provider node at application layer. We designed a new protocol and the appropriate decision algorithms to provide the best multimedia QoE and QoS to the end users participating in the ad hoc network.

We have not found in the related literature any cluster-based ad hoc architecture focused on multimedia streaming.

## 3. Proposed Cluster-Based Architecture

In this section we detail the proposed architecture to build wireless ad hoc clusters for multimedia streaming with service guaranteed. First we will describe the initial state (Init State) of the architecture, which will be used as a starting point for our protocol. Then, we will define the multimedia init profile (MIP), which collects the multimedia information used by the network nodes. Then, we will detail the system processes for the proper operation of the architecture. Finally, the routing algorithm to estimate the most convenient paths for multimedia communications through the cluster will be explained.

### 3.1. Multimedia Init Profile (MIP)

Let multimedia init profile (MIP) be a data structure which represents the multimedia streams delivered through an ad hoc cluster from a source to a destination node. MIP contains a single array with all the information needed to decide the route for each stream. It contains the information of the QoS requisites that should be guaranteed by each cluster node to transmit each type of multimedia stream. Network topological features and the capacity of the nodes in the ad hoc network will determine the most adequate number of nodes and the properties of the MIP available to be selected as an initial configuration by a node. The network topological features are the density of the nodes, their location, space distribution (these data are obtained by using GPS data), obstacles, and possible signal interferences (estimated by using geographical maps or building maps if it is indoors). Other features such as transmission power and coverage area could also be added in future works. From the multimedia streams we have added the type of multimedia stream (video, audio, or both), the used codec, and the QoS requisites (delay, jitter, lost packets, and bandwidth).

The cluster-based architecture uses MIP as a main feature to build the clusters. It groups in the same cluster the nodes with the same MIP under the coverage area. We can adapt the definition of the MIP to each particular case. Moreover, we can define several MIPs for a single cluster. For example, a network with low nodes density in the clusters can have both MIP one for audio transmission and the other for video transmission, but in a network with high density of nodes dedicated only for the video transmission using many types of codecs, they can use several MIPs that will allow them to transmit the streams with different codecs into different clusters. In order to simplify our explanation and the system deployment details, we will assume that all nodes in the ad hoc multimedia cluster share the same MIP, but it can be extended to several MIPs or to MIPs with range of values.

The number of defined MIPs, available to be selected in the system startup, should be wide enough to cover accurately the most common multimedia streams, but it should not be too much to facilitate new node joining and avoid having too many different clusters.

MIP has the following parameters inside: maximum bandwidth, minimum bandwidth, maximum delay, maximum jitter, maximum packet loss, and maximum number of hops. Each MIP has a one byte long hexadecimal code called HCode and an alphanumeric code called ACode, with variable size.

- Maximum bandwidth (MaxBW): this parameter establishes the maximum bandwidth spent by all the multimedia flows processed by the node at the same time. This value represents the whole bandwidth provided by the node for multimedia transmissions with guaranteed service.
- Minimum bandWidth (MinBW): this value describes the minimum bandwidth required to transmit just one multimedia flow. It represents the bandwidth requirements specified by a multimedia codec or a group of multimedia codecs with similar requirements for a single multimedia communication.
- Maximum delay (MaxDelay): this parameter allows knowing the maximum latency value allowed for a multimedia packet across the cluster between the source node and the target node. It represents the maximum guaranteed quality for audio or video transmissions inside a cluster.
- Maximum jitter (MaxJitter): this value indicates the maximum jitter that is considered tolerable for multimedia transmissions inside the cluster.
- Maximum packet loss (MaxLoss): this is the maximum percentage of acceptable lost packets. When this value is exceeded, the target node, the node at the end of the multimedia path, breaks the multimedia transmission and notifies the rest of the nodes in the path because the quality of communication cannot be guaranteed. It is calculated to each one individually. This parameter, together with the maximum delay and maximum jitter, represents the quality of service provided for a real-time communication.
- Maximum hops (MaxHops): this value provides the maximum diameter of the cluster and it can be estimated through the routing table. When a new node joins the network, it will start the connection process trying to connect with a cluster using the same MIP. The system uses the MaxHops value to check that the cluster dimension is always kept under acceptable values for multimedia traffic transmission guaranteeing enough quality of service. A node belonging to a cluster will not accept new joining nodes if it has reached MaxHops.

Table 1 details the list of MIPs set in advance for audio and video transmission. We have tagged an alphanumeric code to each MIP, called ACode, in order to let the node set each MIP and use it in the protocol messages. It is easy to use by the user or by the network administrator when the initial configuration of the node is set. We have also associated a hexadecimal code called HCode, which has a byte size, which will be used in the protocol header when information is exchanged between nodes in the same cluster. Table 1 also shows the QoS parameters associated with each MIP maximum and minimum bandwidth, delay and jitter, the maximum number of hops (MaxHops), and the cluster diameter.

**Figure 1:**
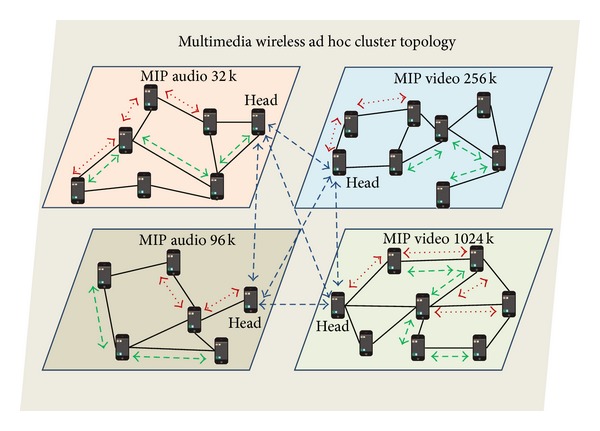
Multimedia ad hoc cluster topology.

Figure 1 shows an example of a multimedia ad hoc network using a cluster-based architecture. All nodes share an area and all are reachable by the other nodes because they are under their wireless coverage area. They are distributed logically in clusters that are specialized in the transmission of similar multimedia streams with similar audio and video QoS parameters. Figure 1 shows how nodes are grouped in 4 clusters, 2 for audio transmission and 2 for video transmission. One video and one audio cluster are dedicated to the transmission of codecs with low bandwidth requirements; the other video and audio cluster are dedicated to the transmission of codecs with higher bandwidth requirements. The head node of each cluster can communicate with head nodes of other clusters in a higher hierarchical level that allows the communication between clusters.

**Figure 2:**
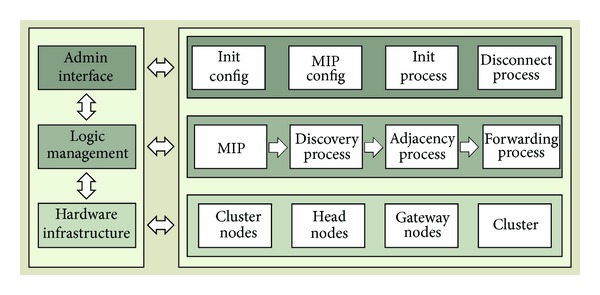
Multimedia ad hoc wireless network architecture elements.

Figure 2 shows the elements of the proposed topology and the relationship between them. The architecture has three levels of operation: hardware infrastructure, logic management, and admin interface. The hardware infrastructure level is formed by different types of nodes (regular cluster nodes, gateway nodes, and head nodes), which build the physical topology, and clusters, which build the logical topology. When new nodes join the network, they have the regular cluster node role. A regular cluster node cannot communicate with nodes from other clusters or with external devices, but with nodes of the same cluster. When a new regular cluster node tries to join the network, it searches nodes under its coverage area. When it receives replies from nodes having the same MIP, the developed protocol will let them exchange information in order to build clusters following the proposed architecture. Each node in the ad hoc network, despite its role, can only belong to a single cluster. First node will be the head node, and it will be responsible for locating and communicating with the head nodes of the other clusters. Gateway nodes have two network interfaces. One interface will be used to connect with the nodes in the ad hoc network and the second interface will be used to connect two with an external network. A node can have both roles: head node and a gateway node.

**Figure 3:**
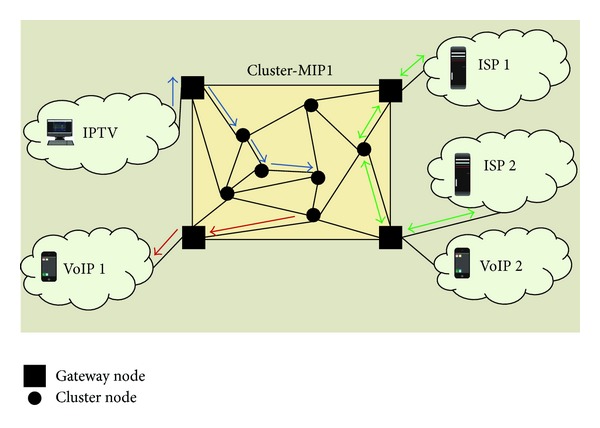
MIP multimedia cluster.

Logic management level defines the elements of the protocol, which will be used to manage the hardware infrastructure elements by gathering the information obtained from the admin interface level. MIP will be used to group the nodes in clusters and assign the cluster to the new nodes. A new node can only be neighbor of a node with the same MIP. All nodes in the same MIP will always have similar features.  Figure 3 shows the internal organization of a cluster. It is formed by a cluster node and a gateway node that use the same MIP. Multimedia streams can be initiated or ended in external multimedia networks like VoIP, IPTV, or ISPs. The connection to external networks is always made by gateway node.

Logic management level also defines the logical processes performed by the nodes automatically as a function of their states and the events given in the network: discovery process, adjacency process, and forwarding process. When a node starts up correctly, it executes the discovery process and seeks other nodes with the same MIP under its wireless coverage area. When it finds other nodes, the adjacency process starts in order to establish a neighborhood between both nodes. The process is repeated every time it finds a new node with the same MIP, allowing the system to build the network clusters. When a cluster is built, it has the capability and resources to retransmit the multimedia streams whose features meet the MIP of the nodes of the cluster. Forwarding process is started when a node starts a new stream query. The query can be started inside the cluster or can be started by another cluster node or by an external network (in this case the query comes from a gateway node). Forwarding process uses the routing algorithm to know the route that should follow the multimedia stream inside the cluster and requests the resource reservation to each node of the route. This process is responsible for establishing the connection between nodes and guaranteeing the QoS required by the MIP during the communication.

The third level is the admin interface level, which allows the interaction between the user and the device. By using a graphical user interface (GUI), node init configuration can be modified, including IP addressing, the MIP to be used by the node, and in case of a gateway node, the communication between the ad hoc network and the external network. Admin interface level is used to manually control the init process and the disconnect process. The user can initialize the node and join or disconnect the node from the ad hoc network. The node can only be configured before the init process starts, so in order to make any change, it is necessary to stop the node, through the disconnect process, perform the appropriate changes, and restart the system with the init process.

### 3.2. System Processes

**Figure 4:**
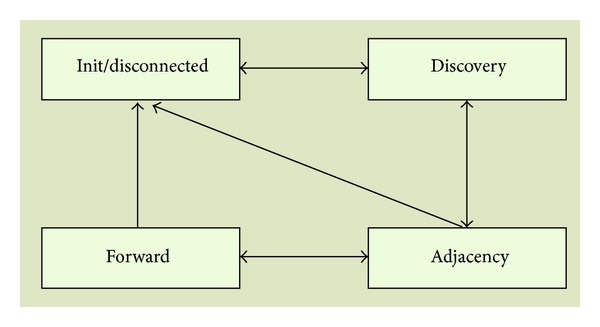
System processes of the multimedia wireless ad hoc cluster architecture.

In order to design the architecture, we propose four basic processes, which correspond to the basic actions of a node inside the ad hoc network. Each process is associated with a set of states and transitions that will be detailed later when the system state machine is explained.  Figure 4 shows the relationship between the processes of the system. Init/disconnect process is the start and end process of the system. It is the only process that requires the user intervention for executing it.

Init process starts the node when the user (or the system) has selected the appropriate MIP. Disconnect process allows the user to leave the network safely (or to restart with a new MIP configuration). Init/disconnect process brings the system to the discovery process, where the node will try to find the nodes in the network with the same MIP. When the node finds another node with the same MIP, and the cluster does not arrive to the maximum number of hops defined by the MIP, the system starts the adjacency process, in which both nodes exchange their network information and lets the new node join the cluster. When a node, belonging to a cluster, receives a query for multimedia stream transmission and checks that it is possible to guarantee MIP requirements, forward process is started.

System processes, with the states of each process, are described in detail next.

(*1)  Init/Disconnect Process.* This process includes the subprocess executed by the node when it joins or leaves the network. This system process includes two states: init state and disconnect state. The node will be in the init state when it is running the init process, and it will be in the disconnect state when it is leaving the network. In the init process the node tries to access the physical network and obtain information about possible neighbors. The init process is divided into three different phases: MIP selection, unicast IP configuration, and group multicast IP configuration. At the first phase, the system allocates the node MIP according to characteristics and the available resources. The MIP of the node can be statically selected by the user, but there is MIP default profile, identified with the HCode value of 0xFF, if no MIP is selected. In the second phase of the init process, the IP configuration of the node is established, including unicast IP address, network mask, and gateway address. The use of a DNS server is optional and it is not required for the normal operation of the protocol. The IP configuration can be manually configured by the user, or may be dynamically obtained by using IETF Zeroconf as defined in RFC 3927. In the next phase of the initialization process the node joins the multicast group matching its MIP. All nodes sharing the same MIP must be listening the same IP multicast group address; thus all nodes in the same multicast group belong to the same cluster. The range of IP multicast group addresses used by the system is 239.100.100.X/24. In this multicast address, the fourth and last byte matches the MIP HCode value. For instance, a node with the MIP 256 K video profile with ACode equal to “V1” and HCode value of 0x41 (decimal value 65) will join the IP multicast group 239.100.100.1.65. Using multicast addresses, each node will communicate exclusively with other nodes with the same profile, without interfering with other nodes in other clusters with different MIP. This system process is also in charge of making the node leave the ad hoc cluster it belongs to. This part of the process occurs when the node is in the disconnect state. The system can reach this state from the other 3 system processes, since the node can leave the system at any time regardless of the assigned state. The node uses the multicast group address of the cluster to notify its neighbors that it is leaving the cluster. Then the neighbors can update their status tables in order to reorganize their forwarding process.

*(2) Discovery Process.* Upon completion of the init process, the node is ready to make the transition to the discovery process. In this process, the node will try to detect the presence of a cluster with the same MIP in order to join it. There are two possible states in the discovery process: discovering state and stand-alone state. When the node accesses the discovery process for the first time, then the system changes to discovering state. This is an active state; while the node stays in this state, it keeps sending discovery messages to the IP multicast group of its MIP. The node waits 60 seconds for replies after sending each discovery message. If no reply is received, during this time interval, another discovery message is sent. Discovering state has a maximum duration of three minutes. If one or more reply messages are received during these 60 seconds after the discovery message is sent, the system changes to the adjacency process. After sending three discovery messages without any result, it changes to the stand-alone state, but still remains in the discovery process in passive mode; that is, the discovery process does not keep sending periodic discovery messages, but the node remains listening for new nodes trying to join the network. If a discovery reply from another node is received, it first checks if the MaxHops of the MIP is not exceeded. If MaxHops is not exceeded, the adjacency process starts.

*(3) Adjacency Process.* This process starts when the above discovery process has detected the presence of one or more nodes with the same MIP. The adjacency process includes join state, associated state, and established state. If a node in the discovery process receives replies from two or more nodes belonging to the same cluster, it will try to establish the adjacency with all detected nodes. If a node receives replies from two or more nodes belonging to different clusters, but all of them are using the same MIP, the system will choose the best cluster and reject other options. The best cluster choice is made by a three-step algorithm, which uses the information included in the discovery reply messages. First, the node estimates the diameter of the cluster if this neighbor is selected. The best selection is the smallest diameter. In case of a tie, the second step comes. The node checks the number of adjacencies of that neighbor and selects the node with the minimum number of established adjacencies in order to distribute the load between different clusters. Finally, if there is a tie in the previous step, it selects the source node of the first received reply. Once the node selects the best candidate, it sends a join message to the selected cluster nodes. When a reply message is received, the node changes to the join state. Then, the new node will receive the information about the cluster characteristics and the topology structure. When the node has the whole information about the cluster, then it changes to the associated state. Finally, the new node sends the information about its resources and availability to the other nodes in the cluster. When all nodes inside the cluster have the same information, the cluster has converged. Then, the new node changes to the established state. In this state, the node is fully integrated in the cluster and it is ready for multimedia transmissions. A node remains in the established state indefinitely until it receives a multimedia transmission request, until the user invokes the disconnect process or until the adjacency is broken. When there is a multimedia request, the forwarding process starts.

*(4) Forwarding Process*. This process is in charge of the multimedia traffic transmission. Inside the forwarding process we can find two different states: queued state and forwarding state. The forwarding state can be initiated only when the node has successfully completed at least one valid adjacency with a node. Multimedia requests could be originated by the node, for example, a request for audio or video communication performed by the user interface, other adjacent nodes, or a gateway node from external networks. When a multimedia request is processed, regardless of the origin, available bandwidth resources at the node are checked. If the node has enough available resources, the node changes to the forwarding state, makes a temporary reservation of resources for the transfer, and notifies the origin node that it is ready for transmission. When all nodes in the path from the source to the target node confirm they have made the resource reservation, the source sends a confirmation message to the nodes in the path to allocate permanently the reserved resources for the multimedia flow. Then, the nodes change their state to the forwarding state and the multimedia transmission takes place. When a node in the multimedia flow path does not have enough resources for the multimedia connection request (e.g., there is not enough available bandwidth) the node changes to the queued state. The node in queued state informs the origin of the multimedia request that it cannot process this request, but it will keep it queued. Then, the source node can wait until the bandwidth resources are released or, if there is some alternative route provided by the routing algorithm, it can cancel the current request and try to establish a new communication using a new path. When the forwarding process for multimedia transmission ends successfully, the node changes to the established state.

### 3.3. Routing Algorithm

Source node (SN) is the node belonging to a cluster that requests a multimedia connection. The request can be performed by a user through the graphical user interface or from external networks (in this case the source node is a gateway node). Target node (TN) is the destination node of the multimedia connection, which will receive and process the multimedia streams. It can be a regular node or a gateway node.

Every node has its neighbors table, which is built and maintained through the adjacency processes, and the cluster topology database which is built using the topology information received from its neighbors. When a SN starts a multimedia transmission, the routing algorithm uses the multimedia streaming bandwidth requirements and the topology information of the nodes inside the cluster. The estimations to determine the route, including the nodes that will forward the multimedia streams inside the cluster, are performed by the SN. The routing algorithm selects as the first hop the node that is the closest (in terms of number of hops) to the TN. When there is a tie, the node with the oldest adjacency will be selected. Selected node is called forward node (FN). FN will estimate the path to the TN using the same process, so it will obtain the second hop in the route to the TN. This process is repeated till TN is achieved. This information is saved in the MEDIA_ROUTE parameter, which will be used by the SN in the resource reservation request in order to guarantee the transmission quality. The resource reservation request is firstly sent to the first FN, which will check if it has enough available resources. If it meets the requirements, it uses the information included in the MEDIA_ROUTE parameter of the message to forward it to the next hop. This process is repeated in each node of the route till it reaches the TN. If the TN receives the request, it means that the cluster has enough resources to perform the multimedia communication meeting MIP requisites, so it replies with a confirmation message that will follow the same route in order to confirm the resource reservation in each node. When the confirmation message reaches SN the multimedia communication starts.

In case of not having enough resources when a node belonging to a route does not have enough resources, the request is included in the queue of this node till it has enough available resources. If the SN receives neither a confirmation reply nor a queue request in 30 seconds (e.g., because a node left the cluster suddenly), it sends a message containing the route verification, which uses MEDIA_ROUTE parameter, to the TN.

Nodes keep updated their neighbor table by sending keepalive messages to all their neighbors and waiting for a reply in less than 10 seconds. If during this process a node detects a topology change, it will send an update message to the rest of the nodes in the cluster to let them update their tables. Both, SN and TN, are able to stop the multimedia streams by closing the communication. They will notify the rest of the nodes of the route that they have to liberate the reserved resources.

## 4. Finite-State Machine

**Figure 5:**
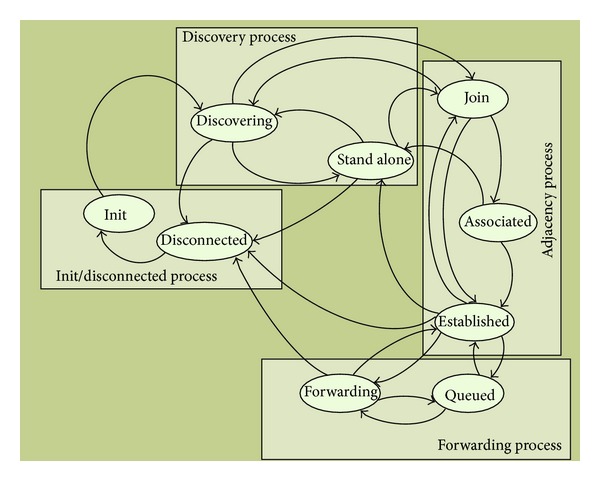
Finite-state machine for multimedia wireless ad hoc cluster architecture.

Figure 5 shows the system finite-state machine. We can see its different states and the transitions between states. In this section we describe each state of the system and the conditions and events that will make the node change from one state to another inside a process.

The processes included in Figure 5 are the following ones.

*(i) Init State.* This is the initial state of the node during the init process. There are two possible ways to access the init state: first, when the node starts for the first time and, second, when the node is rebooting. There is only one possible transition from the init state to the discovering state. This transition is made when the node has initialized correctly; that is, when the whole information has been obtained from the MIP, the IP settings are correct, and the network connection is active. There are several events that may cause the init process to fail: an IP address conflict with another node in the wireless network, the wireless network connection being not enabled, or when it is not possible to join the IP multicast group. When an error event happens in the boot process, the system remains inactive in the init state for 120 seconds before it tries again to initialize the system. 

*(ii) Discovering State.* In this state the node has not yet established any adjacency and it is looking for a neighbor by sending discovery messages. The first time the system makes the transition to the discovering state is when, being at the init state, the system initialization has been completed successfully. The node can also change to the discovering state from the stand-alone state. It happens when the system has remained in the stand-alone state for 12 minutes and no discovery message has been received from other nodes. Finally, there could be a transition to the discovering state from the join state when the adjacency fails in the adjacency process. While the node remains in the discovering state a discovery message is sent every 60 seconds to the IP multicast address of the MIP. The maximum number of discovery messages is set to 3. From the discovering state there is a transition to the join state when the node receives a discovery confirmation message. The waiting time for discovery confirmation messages is set to 60 seconds. Upon finishing 60 seconds the node gathers all received messages and processes them as explained before. Then, there is a transition to the join state. After three times of 60 seconds without receiving any discovery message, a transition to the stand-alone state is made. 

*(iii) Stand-Alone State.* The node reaches this state when the discovery process has not found any valid node, and thus cluster, to join. Then, the node remains isolated from the remaining nodes and it does not establish any adjacency. There are two possible ways to arrive to the stand-alone state: first, when the node is in the discovering state, as described above, and, second, from the established state (it happens when the node has just one established adjacency and it is broken because the neighbor node has left the network). A node assumes that its neighbor is down when it receives a leaving notification or when it has not received any response message during 10 seconds (e.g., after a keepalive message has been sent or in the path verification subprocess that takes place in the forwarding process). There are three admissible transitions from the stand-alone state: discovering state, join state, and disconnecting state. If the node receives a valid discovery message from another node while it stays at the stand-alone state, then the system replies with a discovery confirmation message in order to offer a new adjacency. Then, if it receives a join request message, it will answer with a join acknowledgment message and the system automatically changes the status to join state and the adjacency process starts. If the node remains in the stand-alone state for 12 minutes and no message has been received from another node, it makes a transition to the discovering state. Then, the discovering process starts again an active search for neighbor nodes. Finally, through the intervention of the user, the system can make a transition to the disconnecting state in order to close the connection and leave the network or to restart because some of the values of the initialization process have been changed. 

*(iv) Join State.* This state is the starting point of the adjacency process. The nodes have not yet shared any information from the neighbor tables but they want to build a new adjacency with the discovered node because it has the same MIP. The system can achieve the join state from three different states: discovering state, stand-alone state, and established state. A transition from the discovering state is made when the node has received at least a confirmation of the discovery message and the acknowledge join message. The transition from the stand-alone state takes place when the node has received a new discovery message and a join request message. Finally, the transition from the established state to join state occurs when the node has already one or more established adjacencies and it receives a new discovery message from a new node requesting a new adjacency. The regular next step from the join state is the associated state. It occurs when both nodes have exchanged the whole information in its neighbor tables and the routing database. If the transition to the associated state cannot be completed, because the received information is inconsistent or incomplete, the system will make a transition to the discovering state (if it is the first adjacency) or to the established state (if there are other established adjacencies). A node can establish adjacencies with two or more nodes. 

*(v) Associated State.* This is a transient state. Both nodes have exchanged the neighbor tables and the routing database, but they have not yet confirmed the integration of the new node at the cluster. This state is reached from the join state as described above. From the associated state the node can make two transitions: towards the established state and to the stand-alone state. The transition to the established state will occur when the new node receives the cluster acceptance notification. The transition to the stand-alone state takes place when the node is not accepted and there are no other established adjacencies. 

*(vi) Established State.* At this state the node has established at least a valid adjacency and it is integrated inside the cluster. This is the regular operation mode for a cluster node when no multimedia traffic stream is transmitted through the cluster. In the established state, the node holds a neighbor table with the information about the neighbors and routing database with the cluster topology. The node needs this information to reach other nodes in the cluster and to calculate the best route based on hop count and multimedia available resources. The established state can be activated by a transition from the associated state when a new adjacency is established, from the join state when the an adjacency fails, but there are other active adjacencies in the node, from the forwarding state when a multimedia communication using that node finishes or from the queued state when the resource request remaining in queue is canceled. Possible transitions that can be made from the established state are to the join state, when the node receives a new discovery message, to the stand-alone state, when the last established adjacency in the node is broken, to the forwarding state, when a multimedia transmission request is received and there are enough resources to process it, to the queued state when the node receives a request for multimedia transmission but there are not enough resources to process it at that time, and, finally, to the disconnecting state due to the user intervention when he/she wants to disconnect or reboot the node. 

*(vii) Forwarding State.* In this state the node is processing and transmitting multimedia packets for every received resource reservation request. This state is reached from the established state when the first request for resource reservation is received and completed successfully or, from queued state, when a queued resource request can be satisfied because the node has released enough resources. When the last active multimedia stream on the node finishes its transmission, the system makes a transition to the established state and it remains listening to new requests. If the node receives a new resource reservation request and the needed resources are not available, then the system changes to the queued state. Finally, if the user wants to abort the active multimedia connections in the node in order to reboot or to close the node, it makes a transition to the disconnecting state, but first it notifies it to the source node and target node of each active communication. 

*(viii) Queued State.* The system uses this state when a node is working properly inside the cluster and receives a new multimedia request but it cannot be processed because it has exhausted their bandwidth resources. Queued state can be reached through a transition from the established state or the forwarding state when it receives a new stream request. The node leaves the queued state when it has released enough resources to process the request and it makes a transition to the forwarding state or established state. If the resource request is canceled and there are other active multimedia streams on the node, then a transition to the forwarding state takes place. But if there are no other multimedia streams processed at the same time, then the transition is made to the established state. User can close or restart the node from the queued state making a transition to the disconnecting state. 

*(ix) Disconnecting State*. The node is in this state when the system is shutting down or rebooting, for example, to update the values of its initial configuration, such as the MIP or the IP settings. The system can change to the disconnecting state by the user intervention from several states: discovering state, stand-alone state, established state, queued state, and forwarding state. When the system changes to the disconnecting state the established adjacencies are checked. If there are adjacencies, a notification message is sent to every neighbor in order to let them update their neighbor tables and forward the information to the other nodes in the cluster. If there are active multimedia transmissions, the node notifies the source node and the target node in order to let them cancel the transmission. If the node is restarting, a transition is made from the disconnecting state to the init state.

## 5. System Performance Study

When multimedia streams are sent through ad hoc wireless networks, the bandwidth and the logical topology characteristic requirements should be adjusted as a function of the type of traffic, audio or video, and the codec used for the transmission.

We have deployed our architecture with the aim of measuring the delay and jitter parameters when several multimedia streams in different wireless ad hoc cluster topology configurations are set up. Obtained results will allow us to validate our protocol and architecture proposal, which groups the nodes in clusters based on the MIP. Nodes are classified and clustered based on their capacity to support different types of multimedia streams. Because we wanted to avoid any dependence with the devices characteristics, we used the same hardware configuration for all devices. They had Intel Core 2 Quad Processor working at 2.50 GHz with 2 GB RAM. These devices were connected through a wireless interface, which used IEEE 802.11 g standard. The wireless channel used to perform our test bench was 2.412 MHz.

The parameters of the cluster topology, such as the diameter, are limited based on the type of multimedia stream that is going to be used. The protocol allows several simultaneous multimedia streams guaranteeing the required resources for each one of them in their respective cluster.

We have selected the most appropriated MIPs taking into account the most used video codec characteristics. With the objective of maintaining equilibrium between the flexibility of the options and maintaining a reduced number of profiles, we have defined 3 MIPs for video in our test bench. The values assigned to each MIP are based on our studies previously performed in [21]. Each node in our test bench has any of these MIPs configured before joining the network.

### 5.1. Codecs Comparison

**Figure 6:**
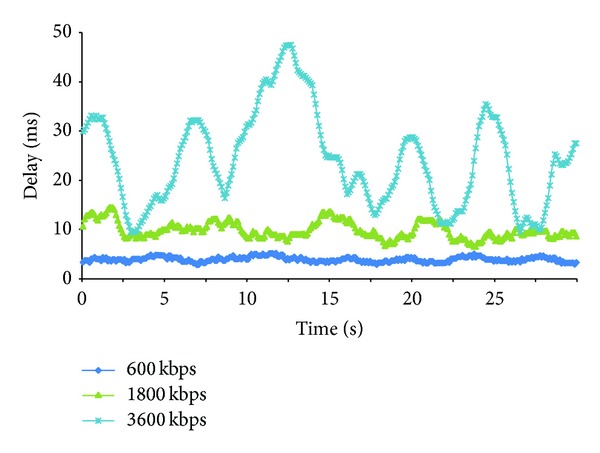
Delay of different streams using different codecs.

In order to compare the multimedia stream behaviour, we have selected three video codecs using 600 Kbps, 1800 Kbps, and 3600 Kbps bandwidth consumption. They correspond to the MIPs V2, V3, and V4 in Table 1. We have analyzed their behaviour when they are being streamed over the same cluster topology and with the same experimental conditions, so the differences in the results are only caused by the codecs characteristics used by each one of the multimedia streams.  Figure 6 shows the delay obtained when those three video codecs are streamed during 30 seconds. In order to provide a graphical representation, we compute the average delay of the last 20 received packets, estimating the value in 100 milliseconds intervals. With *X*_*i*_ being the delay of a single packet, our average delay is given by:

(1) Yi=∑j=ii+20Xi20.

Figure 6 provides *Y* as a function of the time. We can observe that 3600 Kbps has higher delay and has higher delay variation. 1800 Kbps and 600 Kbps are more stable. The one that provides lower values is 600 Kbps.

We have also performed a statistical analysis in order to interpret the results. In order to determine whether the observed differences in the delay are random or are caused by intrinsic characteristics of the codecs, we have defined the following null hypothesis *H*_0_. There is no difference between the average delay obtained by the three codecs with bandwidths of 600 Kbps, 1800 Kbps, and 3600 Kbps.  Table 2 shows the estimations performed for each codec. *N* is the number of samples, *μ* is the average score, *σ* is the standard deviation, Min is the minimum score, Max is the maximum score, and Conf. Int. is the confidence interval. In order to perform the statistical analysis, we have used a confidence level (*α*) of 0.01, with a confidence interval of 99%. The results show that the average delay value of each codec is outside of the confidence interval obtained for all codecs in all analyzed cases, so we can reject the null hypothesis with *P* < 0.01. The highest value has been obtained for 3600 Kbps in all cases, while the lowest value has been obtained for 600 Kbps in all cases. We can conclude that the behaviour of a multimedia stream when using the same cluster topology is different and depends on the bandwidth required by the codec, so we have to use a different treatment. We have also observed that lower bandwidth consumption provides lower delay values with higher confidence.

**Figure 7:**
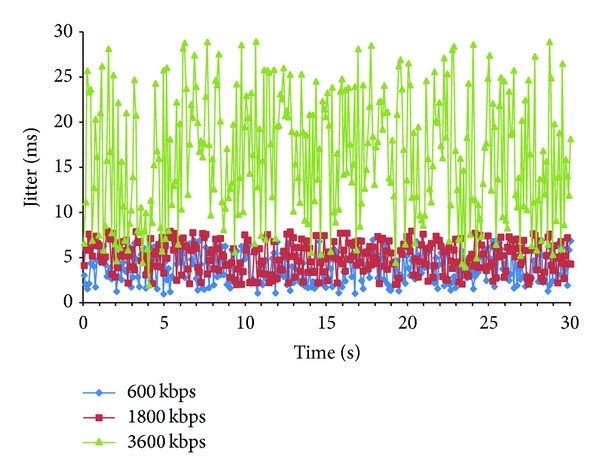
Jitter of different streams using different codecs.

Figure 7 shows the results obtained when jitter is measured as a function of the used codec during 30 seconds. Jitter values are the average jitter values of the last received samples for the three multimedia streams using the same cluster topology. The three streams use the same number of hops (2 hops). We have observed that the jitter is quite higher for the codec with higher bandwidth consumption (3600 Kbps), while it remains quite stable and considerably lower for 1800 Kbps and 600 Kbps.

The statistical analysis provided in Table 3 shows that there is a significant difference between the codec with 3600 Kbps and the other codecs, obtaining *μ* and Max (ms) values 3 times higher. There is also a significant difference with a value of *α* = 1, between 600 Kbps and 1800 Kbps codecs.

### 5.2. Hops Comparison

**Figure 8:**
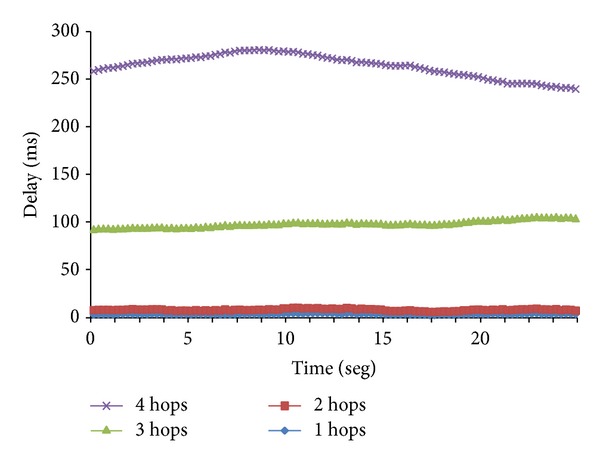
Delay of a multimedia stream of 600 kbps for different cluster diameters.

We performed the following test with the aim to show how a multimedia stream has different quality of service values as a function of the number of hops in the wireless ad hoc cluster. In order to perform this test we have selected a codec with an average of 600 Kbps and we have tested it in four topologies with different number of hops inside the cluster.  Figure 8 shows the obtained delay as a function of the number of hops. We have observed that 1 and 2 hops do not increase the delay much, but it is considerably increased in three hops and hugely increased in 4 hops. Delay values are not increased proportionally with the number of hops.

We have also performed a statistical analysis based on the null hypothesis *H*_0_. There is no difference in the delay average when a multimedia stream of 600 Kbps is being transmitted over several cluster ad hoc networks with diameters 1, 2, 3, and 4 hops.  Table 4 details the estimated values for all obtained data. The estimated parameters are the same as the ones provided for Table 2. We have selected a confidence level (*α*) of 0.01, with a confidence interval of 99%. After obtaining these results we can discard the null hypothesis and affirm that the delay of a multimedia stream in a cluster ad hoc topology depends on the number of hops between the source node and the target node. We have also observed that the main difference is between 2 hops and 3 hops.

**Figure 9:**
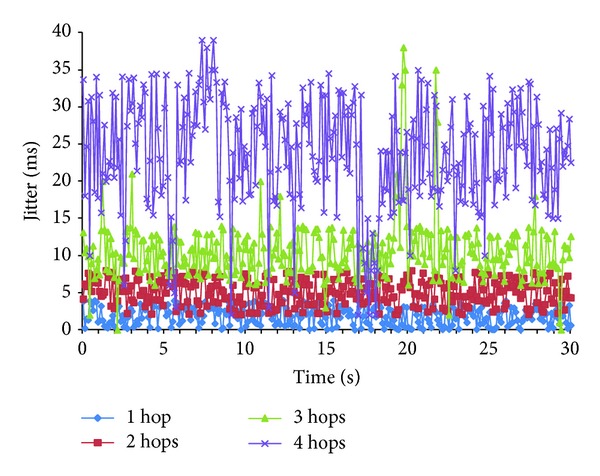
Jitter of a multimedia stream of 600 kbps for different cluster diameters.

Figure 9 shows the measurements gathered for the jitter as a function of the number of hops in the cluster when 1800 Kbps multimedia stream is used. It shows a 30 seconds interval. We have observed that the highest values are obtained for 4 hops. The difference with the rest of cases is high. One hop has the lowest jitter values.

We have performed the statistical analysis of the results with *α* = 1 (see Table 5). We can check that there is a significant difference when the number of hops is increased. Three hops doubles 2 hops values and 4 hops doubles 3 hops values. We can conclude that the jitter values directly depend on the number of hops in the cluster topology.

## 6. Conclusion

In this paper, a new architecture for ad hoc wireless networks has been proposed. It is a cluster-based architecture and it uses QoS profiles to optimize multimedia traffic. The architecture provides a flexible solution with the ability to guarantee the quality of multimedia communication over the ad hoc wireless network. It is able to adapt to many physical network configurations through the suitable selection of the multimedia init profiles (MIPs). The paper shows how QoS parameters and the multimedia codec characteristics affect the topology of the cluster. Moreover, the cluster diameter affects severely the delay and jitter. The proposed architecture provides a control mechanism to build the appropriate topology for each cluster. Furthermore, the system uses a resource reservation scheme to guarantee the quality of the multimedia streams.

In future works we will integrate some mechanisms to allow the system to adapt very fast to spatial changes and node mobility. Moreover, we will add security to the communications through authentication integrity and confidentiality techniques. Our final purpose is to deploy the proposed architecture in a real environment to provide multimedia streaming in wireless sensor networks [22].

## Figures and Tables

**Table 1:** Defined MIP list for the practical implementation of the architecture.

MIP	ACode	HCode	MinBW	MaxBW	MaxDelay	MaxJitter	MaxHops	MaxLoss
Audio 32 K	A1	0x01	8 Kbps	32 Kbps	50 ms	20 ms	6	0.5
Audio 64 K	A2	0x02	8 Kbps	64 Kbps	100 ms	40 ms	6	0.5
Audio 128 K	A3	0x03	16 Kbps	128 Kbps	150 ms	40 ms	5	0.5
Audio HQ	A4	0x04	32 Kbps	1024 Kbps	150 ms	40 ms	4	0.5
Video 256 K	V1	0x41	64 Kbps	256 Kbps	100 ms	20 ms	4	1
Video 1024 K	V2	0x42	128 Kbps	1024 Kbps	150 ms	40 ms	4	1
Video 2048 K	V3	0x43	256 Kbps	2048 Kbps	200 ms	40 ms	3	1
Video HQ	V4	0x44	1024 Kbps	20 Mbps	200 ms	40 ms	2	1
Default	Default	0xFF	56 Kbps	1 Mbps	200 ms	40 ms	4	1

**Table 2:** Statistical values of the delay of different streams using different codecs.

Video codecs	Parameters						
	*N*	*µ* (ms)	*σ* (ms)	Min (ms)	Max (ms)	Conf. Int. (ms)	
600 Kbps	300	3.96	0.51	2.86	5.23	3.88	4.03
1800 Kbps	300	9.95	1.72	6.62	14.52	9.69	10.20
3600 Kbps	300	24.27	9.57	9.52	47.58	22.84	25.70

**Table 3:** Statistical values of the jitter of different streams using different codecs.

Video codecs	Pararameters						
	*N*	*µ* (ms)	*σ* (ms)	Min (ms)	Max (ms)	Conf. Int. (ms)	
600 Kbps	300	3.94	2.91	1	7	3.68	4.19
1800 Kbps	300	5.11	1.72	2	8	4.85	5.37
3600 Kbps	300	16.26	48.17	2	29	6.21	17.29

**Table 4:** Statistical values of the delay as a function of the diameter.

HOPS	Parameters						
	*N*	*µ* (ms)	*σ* (ms)	Min (ms)	Max (ms)	Conf. Int. (ms)	
1	100	3.96	0.51	2.86	5.23	3.88	4.03
2	100	4.40	0.52	3.02	6.21	4.32	4.47
3	100	96.66	5.60	85.02	106.11	95.83	97.47
4	100	165.38	72.162	158.08	1783.02	154.86	175.90

**Table 5:** Statistical values of the jitter as a function of the diameter.

HOPS	Parameters						
	*N*	*µ* (ms)	*σ* (ms)	Min (ms)	Max (ms)	Conf. Int. (ms)	
1	300	1.99	1.23	0	6	1.80	2.17
2	300	5.11	1.72	2	8	4.85	5.37
3	300	10.56	4.28	0	38	9.92	11.19
4	300	23.71	7.57	2	39	22.58	24.84
